# Glycan Epitopes and Potential Glycoside Antagonists of DC-SIGN Involved in COVID-19: In Silico Study

**DOI:** 10.3390/biom11111586

**Published:** 2021-10-27

**Authors:** Meina Gao, Hui Li, Chenghao Ye, Kaixian Chen, Hualiang Jiang, Kunqian Yu

**Affiliations:** 1 School of Chinese Materia Medica, Nanjing University of Chinese Medicine, Nanjing 210023, China; 20193142@njucm.edu.cn (M.G.); kxchen@simm.ac.cn (K.C.); hljiang@simm.ac.cn (H.J.); 2 Drug Discovery and Design Center, State Key Laboratory of Drug Research, Shanghai Institute of Materia Medica, Chinese Academy of Sciences, Shanghai 200031, China; lihui1@shanghaitech.edu.cn; 3 University of Chinese Academy of Sciences, Beijing 100049, China; 4 Shanghai Institute for Advanced Immunochemical Studies, School of Life Science and Technology, ShanghaiTech University, Shanghai 200031, China; 5 Department of Chemistry, Shantou University, Shantou 515063, China; 18chye@alumni.stu.edu.cn

**Keywords:** DC-SIGN, glycan epitopes, carbohydrate recognition mechanism, natural glycoside antagonists, molecular dynamics simulations, COVID-19

## Abstract

Glycosylation is an important post-translational modification that affects a wide variety of physiological functions. DC-SIGN (Dendritic Cell-Specific Intercellular adhesion molecule-3-Grabbing Non-integrin) is a protein expressed in antigen-presenting cells that recognizes a variety of glycan epitopes. Until now, the binding of DC-SIGN to SARS-CoV-2 Spike glycoprotein has been reported in various articles and is regarded to be a factor in systemic infection and cytokine storm. The mechanism of DC-SIGN recognition offers an alternative method for discovering new medication for COVID-19 treatment. Here, we discovered three potential pockets that hold different glycan epitopes by performing molecular dynamics simulations of previously reported oligosaccharides. The “EPN” motif, “NDD” motif, and Glu354 form the most critical pocket, which is known as the Core site. We proposed that the type of glycan epitopes, rather than the precise amino acid sequence, determines the recognition. Furthermore, we deduced that oligosaccharides could occupy an additional site, which adds to their higher affinity than monosaccharides. Based on our findings and previously described glycoforms on the SARS-CoV-2 Spike, we predicted the potential glycan epitopes for DC-SIGN. It suggested that glycan epitopes could be recognized at multiple sites, not just Asn234, Asn149 and Asn343. Subsequently, we found that Saikosaponin A and Liquiritin, two plant glycosides, were promising DC-SIGN antagonists in silico.

## 1. Introduction

Glycosylation is a common post-translational modification that affects a wide range of physiological activities. Glycan-binding proteins (GBP) recognize the majority of these glycans. One GBP is DC-SIGN, which belongs to the C-type lectin superfamily. Dendritic cells, macrophages, neutrophils, and other antigen-presenting cells are the major cells expressing DC-SIGN [1]. DC-SIGN is a 404 tetrameric transmembrane protein with residues 1–95 located in the cytoplasm, 96–257 in the seven neck repeat regions outside the membrane, and 263–404 in the carbohydrate recognition region [2]. DC-SIGN must work in tandem with Ca^2+^. The activation of APC endocytosis via DC-SIGN promotes virus transfection, which is considered one of the causes of systemic organ infection. An EPN motif in the DC-SIGN carbohydrate recognition domain recognizes *L-Fucose* (Fuc), *D-Mannose* (Man), *Glucose* (Glc) and *N-Acetylglucosamine* (GlcNAc) via octahedral coordination bonds with Ca^2+^ roughly at the millimolar level [3]. The order of binding affinity is *L-Fucose*, followed by *Mannose*, *N-Acetylmannosamine* (ManNAc), *Glucose* and *N-Acetylglucosamin* [4,5]. DC-SIGN could recognize polysaccharides such as Lewis X, high oligomannose, and LPS [6,7,8]. Moreover, DC-SIGN recognizes a wide range of viral glycoproteins, including HIV gp120 [9], Ebola glycoprotein [10], Hemagglutinin [11], and dengue virus glycoprotein [12].

DC-SIGN (CD209) and L-SIGN (CD299) can bind to the sugar chain of SARS-CoV-2 Spike protein, triggering antigen-presenting cell endocytosis and immune escape or systemic pulmonary infection [13]. The SARS-CoV-2 virus is a coronavirus with a highly glycosylated protein outside of the membrane. Along with its 1273 amino acids, the Spike glycoprotein contains 22 N-linked glycosylation sites and several O-linked glycosylation sites [14,15,16]. However, there is a discrepancy in *O-glycosites* that the two groups obtained 17 and 25 sites, respectively [17,18]. Previous studies have demonstrated that DC-SIGN recognizes glycoform at different Spike locations. The NMR method revealed that DC-SIGN recognizes the Spike protein’s Lewis X and LNDF sugar motifs [19]. Flow cytometry was used to confirm that DC-SIGN adhered to the Asn149 glycans in the NTD domain [20]. Electron microscopy directly showed that DC-SIGN recognizes multiple Spike sites [21]. Recently, it was proposed that DC-SIGN interacted with high oligomannose at Asn234 [22]. Microarray glycan assays revealed that DC-SIGN recognizes the complex type of glycans [23]. According to a recent study, one Spike can attach an average of 3.6 DC-SIGNs [24]. The variation in DC-SIGN recognition may be explained by physiological heterogeneity. The explanations are usually classified into two categories: first, glycosylation is heterogeneous. Glycoproteomics reveals the presence of several glycoforms at a single glycosylation site [25]. Second, the glycans on the protein surface undergo constant conformational changes in the solution. Glycans, as demonstrated by molecular dynamics simulations, may shield the surface and expose different glycan epitopes at different states [26]. Monoclonal antibodies against DC-SIGN, as expected, can inhibit DC-SIGN binding to Spike and reduce pseudo-virus transmission in vitro. It is vital to investigate the mechanism of DC-SIGN to develop new COVID-19 therapies [27,28].

Using electron microscopy, DC-SIGN has been reported to bind SARS-CoV-2 Spike glycoproteins, but the molecular mechanism of DC-SIGN interacting with the glycoprotein is yet to be demonstrated [21]. Although the GLYCAM force field can perform the dynamics of glycoproteins, investigating the mechanism by which DC-SIGN recognizes glycan epitopes on the protein surface remains a challenge [29]. Starting with known DC-SIGN antagonists could be an alternative strategy to demonstrate the mechanism.

Since the beginning of this century, the intervention of DC-SIGN recognition has yielded some results [30]. Related research reports that antagonists can be categorized into three groups. First, the analogs of natural polysaccharide structures, such as mannobiose derivatives [31] and polysaccharide polyman26 [32]. Second, non-carbohydrate inhibitors, such as quinoxalinones which can bind to DC-SIGN block the recognition [33]. Third, glycan-modified macromolecular materials and mannose-modified nanoparticles can compete with the virus to bind DC-SIGN [34]. All of these factors contribute to the discovery of the carbohydrate recognition mechanism.

In this study, we used molecular dynamics to demonstrate how DC-SIGN binds to monosaccharides. We discovered three potential pockets that hold different glycan epitopes by performing molecular dynamics simulations of previously reported oligosaccharides. Based on our findings and previously described glycoforms on the SARS-CoV-2 Spike, we predicted the potential glycan epitopes for DC-SIGN. Finally, we discovered natural glycosides in traditional Chinese medicine that could bind to DC-SIGN. We performed preliminary validation using molecular dynamic simulations.

## 2. Materials and Methods

### 2.1. Ligand Preparation

The GLYCAM website (https://dev.glycam.org/cb/, accessed on 15 July 2021) is used to construct the structure of certain commonly used glycosides [35]. A total of five molecular oligosaccharide chain structures were constructed, namely Manα1,2[Manα1,6]Man; Manα1,2Manα1,2Man; Manα1,2Manα1,3Man; Glcα1,3Glcα1,3Glc; and GlcNAcβ1,4[Fucα1,6]GlcNAc. The remaining oligosaccharides were synthesized using the crystal structure of the existing DC-SIGN complex (PDB: 1K9I, 1SL4, 1SL5, 2IT5, 2IT6). The LigPrep module of the Schrodinger suite (Schrödinger release 2020-4: Schrödinger, LLC, New York, NY, USA) was used to complete the glycoside structure preparation before the screening. The ligand was imported to the Schrodinger workspace and the ligand’s 3D structure was generated by adding hydrogen atoms, eliminating salt, and ionizing at pH 7 ± 2. The OPLS3e force field [36] was used for energy minimization using the standard energy function of molecular mechanics, and the RMSD (Root-Mean-Square Deviation) was reduced to 0.01 Ǻ to generate low-energy ligand isomers. For each ligand, a maximum of 32 stereoisomers was set.

### 2.2. Protein Preparation and Grid Generation

In this study, the complex structure (PDB: 1SL4) of the DC-SIGN carbohydrate recognition domain (CRD) and Man4 were downloaded from the Protein Data Bank database. The X-ray crystal structure had a resolution of 1.55 Å. The structure of the complex was imported into Schrodinger suite Maestro (Maestro v12.6, Schrödinger, LLC, New York, NY, USA). In addition, the Protein Preparation Wizard [37] tool was used to perform the following operations on the protein: add hydrogen atoms and remove water molecules that form fewer than 3 hydrogen bonds with Man4. The active site grid was generated using the Receptor Grid Generation application in the Glide module (Glide V8.9, Schrödinger, LLC, New York, NY, USA). Glide uses a filter search to locate the ligand in the active site region of the receptor. The shape and characteristics of the receptor were displayed on a grid, providing a more accurate ligand posture score. At the co-crystallized ligand Man4, a grid with a radius of 10 Å was generated. Furthermore, the OPLS3e force field was used in both protein-energy minimization and grid formation.

### 2.3. Molecular Docking

Molecular docking is a structure-based drug design technology that identifies potential interaction modes between proteins and small-molecule ligands. The scoring function predicts the binding affinity between the ligand and the receptor based on the minimum interaction of the ligands. Glide Standard precision (SP) is a docking protocol in the Schrodinger suite Glide module that does not impose any restrictions. We performed Glide Standard precision (SP) docking on the molecules prepared by LigPrep (Schrödinger, LLC, New York, NY, USA) to predict the binding affinity between DC-SIGN and different oligosaccharides. Each ligand can yield up to 10 poses, and the visualization in docking results and oligosaccharide structures were performed by PyMOL (Schrödinger, LLC, New York, NY, USA) and VMD (Visual Molecular Dynamics) [38], respectively.

### 2.4. Molecular Dynamic Simulations

The sugar recognition mechanism of DC-SIGN was studied at the atomic level using molecular dynamics simulation. The study was conducted on a Centos7-based Linux workstation with the CUDA (Compute Unified Device Architecture) version of the Amber 18 software package (AMBER 2018, University of California, San Francisco, CA, USA) for MD simulations. The topology was generated with the tLeap module, and the protein structure was represented by the Amber ff14SB force field [39]. Research has shown that GLYCAM06j force fields have the best binding free energy calculation effect in the simulated Protein–Carbohydrate Complexes systems, followed by GAFF1.7/AM1-BCC [40]. Since most of the natural glycosides are glycoside derivatives rather than standardized glycosides, we finally chose to use the GAFF2/AM1-BCC force field for further simulations instead of GLYCAM force-fields. Therefore, ligand charge, which was considered as the BCC charge, was computed by the antechamber module in AmberTools18 and was defined by the GAFF2 force field [41]. The SPC/E water model was applied to any systems containing the counter-ions Cl^−^ or Na^+^. The systems were first minimized through the following steps before the MD simulations: (1) the 1000 steps of the steepest descent and the 500 steps of the conjugate gradient were submitted for calculation, under the harmonic constraint of 2.0 kcal/(mol·Å2) on heavy atoms; (2) the system was gradually heated to 300 K through 50 ps NVT simulation, under the weak limit of 2.0 kcal/(mol·Å2). A 50 ps NPT equilibrium simulation was performed at 1 atm. The Langevin thermostat and Berendsen barostat were used to maintain the temperature and pressure. In addition, the SHAKE algorithm [42] was applied to all hydrogen atoms in the simulation process with a time step of 2 fs. Finally, each system was subjected to a stimulation lasting up to 500 ns to acquire a stable conformation of the complex in the system.

The *cpptraj* module in the AmberTools18 was used to calculate the root-mean-square deviation (RMSD), the root-mean-square fluctuation (RMSF) values, and hydrogen bonds (H-bonds). The RMSD and RMSF during the equilibrium were calculated using the initial structure as a reference. Furthermore, we statistically classified the hydrogen bonds formed between the oligosaccharides and DC-SIGN in the entire trajectory based on the residue number and calculated the contribution rate of each protein residue to the hydrogen bonds using the following formula:

H-Bond Contribution Rate = *H-Bond*_residue_/*H-Bond*_sum_ (1)

In the formula, *H-Bond*_residue_ represents the total number of hydrogen bonds formed by a residue in the trajectory and *H-Bond*_sum_ represents the total number of hydrogen bonds formed by DC-SIGN and oligosaccharides in the track.

Furthermore, we captured 1 frame every 1 ns and used the *molsurf* program in *cpptraj* module to calculate the solvent-accessible surface area (SASA) where the DC-SIGN protein is covered by oligosaccharide molecules. The following is the calculation method:

*SASA*_cover_ = (*SASA*_protein_ + *SASA*_ligand_ − *SASA*_complex_)/2 (2)

Among them, *SASA*_cover_ represents the interface area of the system. *SASA*_protein_, *SASA*_ligand_, and *SASA*_complex_ represent the *SASA* of protein, and ligand and protein-ligand complex, respectively.

### 2.5. Calculation of Binding Energy/MM-GBSA

The Molecular Mechanics Generalized Born Surface Area (MM-GBSA) method [43,44] is widely used to estimate the relative binding energy of protein-ligand and protein–protein systems. In addition, existing studies have shown that the implicit solvent model MM-GBSA can well simulate the binding free energy of the Protein/Carbohydrate complex system [45]. In the MM-GBSA calculation, the average total free energy (Δ*G*_bind_) of the system was calculated as follows:

Δ*G*_bind_ = *G*_complex_ − (*G*_protein_ + *G*_ligand_) (3)

Δ*G*_bind_ = Δ*G*_MM_ + Δ*G*_sol_ − *T*Δ*S* (4)

Among them, Δ*G*_MM_ is the free energy of molecular mechanics, and Δ*G*_sol_ and *T*Δ*S* are the solvation free energy and entropy contribution, respectively. In addition, the Δ*G*_MM_ value includes van der Waals energy (Δ*G*_VDWAALS_) and electrostatic (Δ*G*_EEL_) energy:

Δ*G*_MM_ = Δ*G*_VDWAALS_ + Δ*G*_EEL_ (5)

The electrostatic solvation Δ*G*_sol_ can be expressed as the free energy of polar solvation (Δ*G*_EGB_) and the free energy of non-polar solvation (Δ*G*_ESURF_) as follows:

Δ*G*_sol_ = Δ*G*_EGB_ + Δ*G*_ESURF_ (6)

We extracted 100 frames from the 18–20 ns dynamic trajectory for the MM-GBSA calculation under the Amber force field. The binding free energy was calculated using MMPBSA.py (the Python version, AMBER 2018, University of California, San Francisco, USA), coded in Ambertools18 software [46]. For the active compounds *Saikosaponin A* and *Liquiritin*, 100 frames from the 498–500 ns trajectory were collected for binding free energy calculation. We also uniformly extracted 500 frames from the entire trajectory and performed energy calculations for the Man4 structure in the crystal structure (PDB: 1SL4). Residue energy decomposed the protein residues within 5 Ǻ around Man4.

## 3. Results

### 3.1. Molecular Dynamics Simulations Accurately Explain DC-SIGN Recognition of Monosaccharides

**Figure 1:**
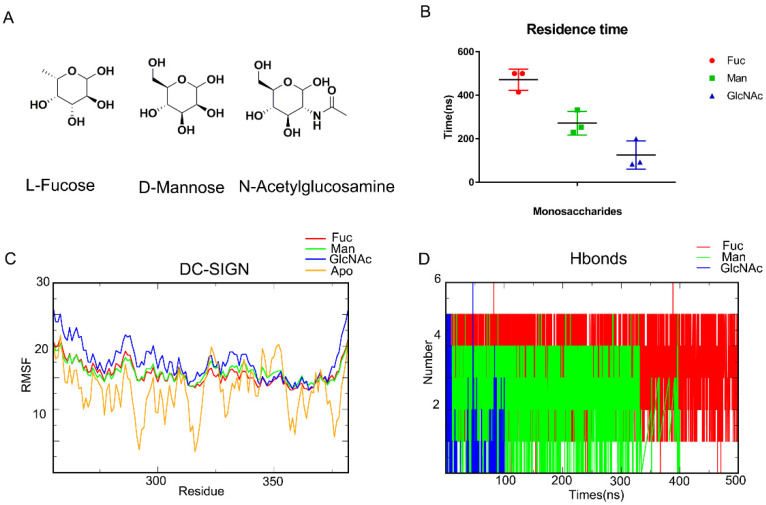
MD simulations demonstrate the recognition of DC-SIGN and monosaccharides. (**A**) *L-Fucose*, *D-mannose*, *N-acetylglucosamine*. (**B**) RMSD about each complex, the Fuc-DC-SIGN last for 500 ns. GlcNAc for the least. (**C**) RMSF of DC-SIGN along Fuc, Man, GlcNAc and Apo trajectories. Bound with monosaccharides would enhance the stability of the loop from Arg344 to Val351. (**D**) Number of Hydrogen bonds in each system. *L-Fucose* bind with DC-SIGN with the most hydrogen bonds. *L-Fucose* in red, *D-Mannose* in green, N-acetylglucosamine in blue.

MD simulation is a common method for capturing the behavior of proteins and other biomolecules with precise temporal resolution and comprehensive atomic detail. We attempted to characterize the mechanism of carbohydrate recognition using MD simulation. Earlier research has shown that *D-Mannose* (Man), *L-Fucose* (Fuc), and *N-Acetylglucosamine* (GlcNAc) can interact with DC-SIGN (Figure 1A). The affinity which is ranked from largest to smallest comprises *L-Fucose*, *D-Mannose*, and *N-Acetylglucosamine*. As a result, we used Glide (Glide V8.9) to construct DC-SIGN-binding monosaccharides conformations (Figure S1). MD simulations of the complexes were performed for 500 ns. Residence times of these three monosaccharides are analyzed (Figure 1B). It showed that Fuc are the most stable. Affinity appeared to be related to residence time. Meanwhile, DC-SIGN remained stable along those trajectories, according to RMSF (Figure 1C). Being bound with monosaccharides would enhance the stability of the loop from Arg345 to Val351. Subsequently, the hydrogen bonds formed from the trajectories were enumerated. The number of hydrogen bonds formed between *L-Fucose* and DC-SIGN was significantly much greater than those formed between *D-Mannose* and *N-Acetylglucosamine* (Figure 1D). Finally, we used MM-GBSA to compute the binding free energy. The binding free energies of *L-Fucose*, *D-Mannose*, and *N-Acetylglucosamine* were found to be −12.3085 kcal/mol, −5.1706 kcal/mol, and −4.0112 kcal/mol, respectively (Table 1). These results demonstrated that MM-GBSA can define the affinity. All of these parameters confirmed that MD simulation is an appropriate method for describing DC-SIGN-recognized monosaccharides.

### 3.2. Glycan Epitopes Bind to DC-SIGN via Different Mechanisms

Monosaccharides and their derivatives can be used as DC-SIGN antagonists. Polyman26, a mannose-modified dendrimer, for example, can block HIV and SARS-CoV-2 transmission via DC-SIGN [21]. THP-1 cells may internalize mannose-modified nanoparticles via DC-SIGN, and monocytes can phagocytize them to induce immune responses [47]. These investigations demonstrated that monosaccharides interact with DC-SIGN and stimulate immunity. It also demonstrated that the Manα1,2Man-decorated gold nanomaterials have a higher affinity than monosaccharides [34]. In addition, recent studies report that Manα1,2Man is a minimal epitope for rational design glycomimetic antagonists [48]. However, another study found that the affinity of Man1,3[Man1,6]Man was greater than that of Man1,2[Man1,6]Man [49,50]. Based on this discrepancy, we hypothesized that various glycan epitopes bind to DC-SIGN via distinct molecular recognition mechanisms. We further postulated that DC-SIGN recognized multiple glycan epitopes via different sites.

**Figure 2:**
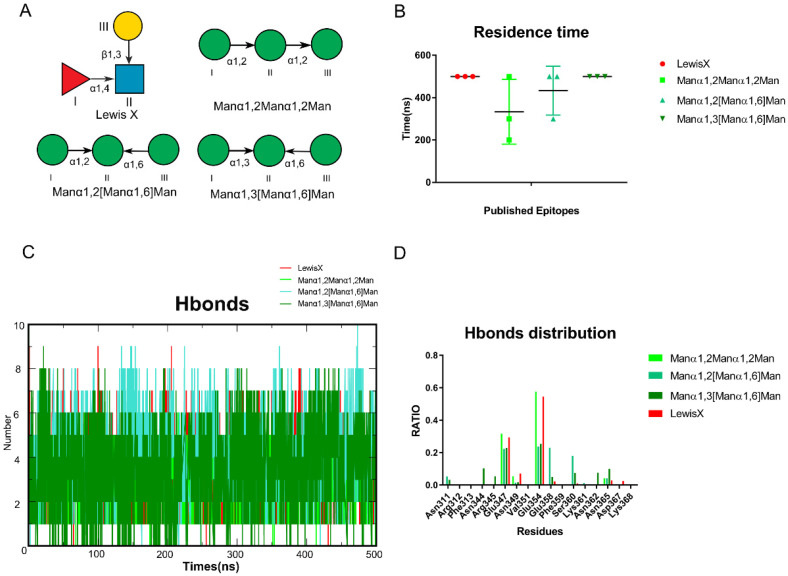
Experiments reported glycan epitopes recognized by DC-SIGN performed through MD simulations. (**A**) Known Glycan epitopes that bind with DC-SIGN.0 LewisX, Manα1,2Manα1,2Man, Manα1,2[Manα1,6]Man, Manα1,3[Manα1,6]Man. (**B**) Residence time of these systems in triple replica. (**C**) The number of hydrogen bonds between the oligosaccharides and protein along the trajectories. Manα1,2[Manα1,6]Man, Manα1,3[Manα1,6]Man formed more than LewisX and Manα1,2Manα1,2Man. (**D**) Four oligosaccharides hydrogen bonds distribution RATIO along each trajectory. The LewisX and Manα1,2Manα1,2Man are alike. Manα1,2[Manα1,6]Man, Manα1,3[Manα1,6]Man shows higher similarity. Lewis X in red, Manα1,2Manα1,2Man in light green, Manα1,2[Manα1,6]Man in green, Manα1,3[Manα1,6]Man in dark green.

To test our hypothesis, we collected published glycan epitopes recognized by DC-SIGN, including Lewis X, Manα1,2Manα1,2Man, Manα1,2[Manα1,6]Man and Manα1,3[Manα1,6]Man [4,19,23] (Figure 2A). Glide docking was used to construct the oligosaccharides that bind to DC-SIGN. The conformations of the oligosaccharides were adjusted so that (1) 3-OH and 4-OH were coordinated with Ca^2+^; (2) the conformations were close to the reported crystal structures as possible (Figure S2). Then, in AMBER, we performed MD simulations of those complexes for 500 ns. To guarantee the reliability of the results, three independent repeats of the MD simulations were performed for each complex and we used the average residence time to assess stability (Figure 2B). The number of hydrogen bonds formed between oligosaccharides and DC-SIGN along all trajectories was calculated using the above data. Lewis X was found to possess the most hydrogen bonds along the trajectories (Figure 2C). We further analyzed the distribution of hydrogen bonds to learn more about them. Glu347, Asn349, Asn365, Asp366 and Glu354 have made significant contributions to all the trajectories. Furthermore, Manα1,2[Manα1,6]Man and Manα1,3[Manα1,6]Man shared Asn311, Glu358 and Ser360. It is worth noting that three more residues, Asn362, Asn344 and Arg345, contributed to the Manα1,3[Manα1,6]Man system (Figure 2D). We validated that various epitopes bind at different sites of DC-SIGN based on hydrogen distribution.

**Figure 3:**
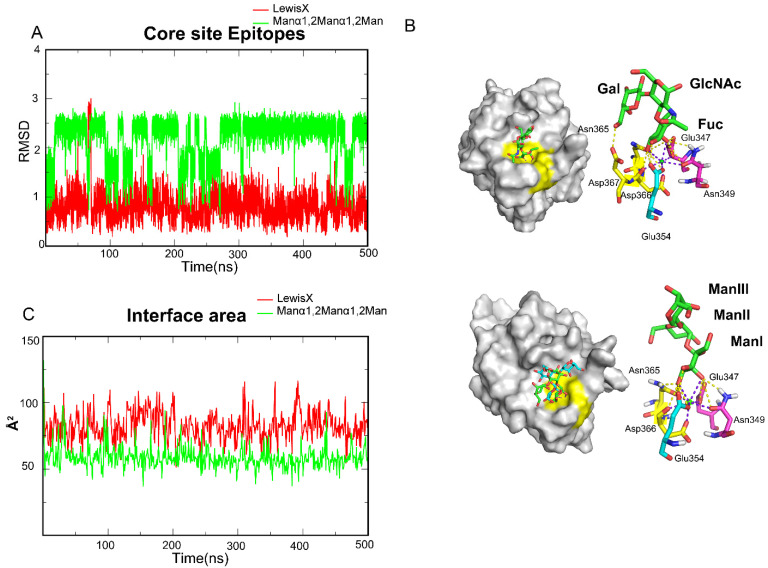
MD simulations showed that DC-SIGN recognizes Lewis X and Manα1,2Manα1,2Man only through the Core site. (**A**) RMSD along one of its trajectories about Lewis X and Manα1,2Manα1,2Man. LewisX kept a single structure along the trajectory. Manα1,2Manα1,2Man showed a conformation change along the trajectory. (**B**) Glu347, Asn349, Glu354, Asn365 and Asp366 fixed the *L-Fucose* and *D-Mannose*. Glu347, Asn349, Glu354, Asn365 and Asp366 formed the Core site. Core sites are shown by yellow. (**C**) Interface area between the oligosaccharides and DC-SIGN along the trajectory. Lewis X is more than a monosaccharide and Manα1,2Manα1,2Man is about monosaccharides. Lewis X in red, Manα1,2Manα1,2Man in light green.

To further investigate the mechanisms, the four oligosaccharides were divided into two groups based on hydrogen bond distribution, of which Lewis X and Manα1,2Manα1,2Man are members. The root-mean-square-deviation (RMSD) values of oligosaccharide atoms relative to the initial binding conformations were measured to assess ligand stability during the simulation period and to study the detailed conformations of those ligands along the trajectories. We found that Lewis X maintained a stable conformation with DC-SIGN throughout the entire trajectory (Figure 3A), in which the EPN motif, NDD motif and Glu354 clamped FucI through 3-OH and 4-OH (Figure 3B). The other two sugars in Lewis X may form hydrogen bonds with the Val351, Lys368 and EFS motif in various frames along the trajectory (Figure 2D). Unlike Lewis X, however, Manα1,2Manα1,2Man has two alternative binding stances on the surface of DC-SIGN (Figure 3A). The ManI is always locked in the two conformations via the EPN motif, NDD motif and Glu354, while the other two Man repeats in the states of binding and dissociation (Figure 3B). In the Manα1,2Manα1,2Man system, only Glu347, Asn349, Glu354, Asn365, and Asp367 form a stable pocket for fixing monosaccharides. To further investigate the electrostatic interaction, we calculated the interface area between the ligand and protein. The area of Lewis X interaction with DC-SIGN is bigger than that of Manα1,2Manα1,2Man, which explains the differences in affinities between the Lewis X and Manα1,2Manα1,2Man systems (Figure 3C). Furthermore, the interface area of Manα1,2Manα1,2Man was found to be comparable to that of monosaccharides (Table S1), reconfirming that monosaccharide is the minimum epitope for DC-SIGN. Based on this, we determined that the monosaccharide-containing site is the critical pocket, dubbed the Core site, for DC-SIGN recognition.

**Figure 4:**
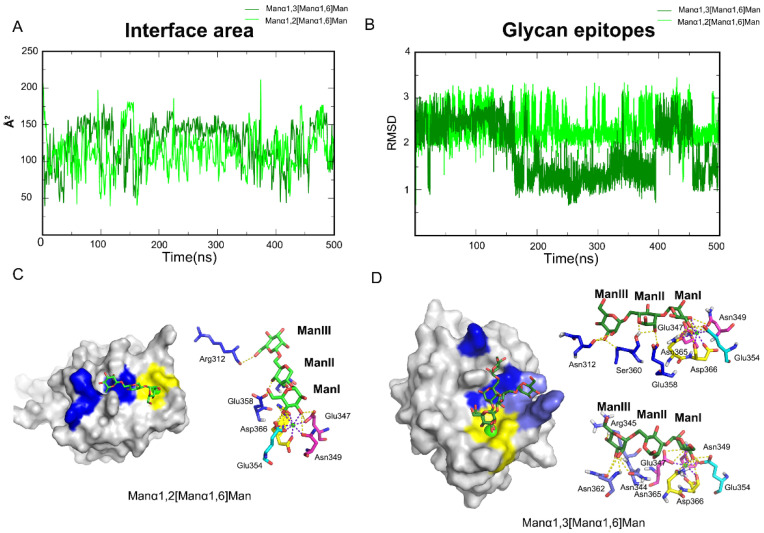
Aside from Core site, Rift and Valley sites on DC-SIGN that held Manα1,2[Manα1,6]Man and Manα1,3[Manα1,6]Man epitopes. (**A**) Interface area between two oligosaccharides and DC-SIGN along the trajectories. (**B**) RMSD of Manα1,2[Manα1,6]Man and Manα1,3[Manα1,6]Man along trajectories. (**C**) Manα1,2[Manα1,6]Man bind modes along the trajectory. EFS motif fixed Manα1,2[Manα1,6]Man through 3-OH and 4-OH in the ManII ring. (**D**) Two conformations of Manα1,3[Manα1,6]Man along the trajectory. EFS motif was consist of Glu358, Phe359, Ser360. Rift site is consist of Arg312, Phe313, Glu358. Valley is consist of Asn344, Arg345, Asn362. One in the Rift site, the other in the Valley. Core site is shown in yellow, Rift site is shown in blue; Valley is shown in purple-blue.

The other group was made up of Manα1,3[Manα1,6]Man and Manα1,2[Manα1,6]Man. Aside from the Core sites, the two oligosaccharides interact with additional residues along the trajectories (Figure S3). Because the two oligosaccharides differ only at a linker, we calculated the interface area first. As expected, the interface areas of the two systems were nearly equal (Figure 4A). During the simulations, Manα1,2[Manα1,6]Man maintained a stable conformation on the oligosaccharides atom RMSD. However, Manα1,3[Manα1,6]Man revealed an additional potential binding site and a cycle between them (Figure 4B). The complex structures along the trajectories were analyzed to locate the pockets.

The 3-OH and 4-OH in ManI were fixed by the Core site for Manα1,2[Manα1,6]Man. Glu358 and Ser360 fixed ManII via 3-OH and 4-OH. ManIII formed hydrogen bonds with Asn311 and Arg312 (Figure 4C). The surface shape of DC-SIGN, the EFS motif and Asn311 combined to form a stable site, like a Rift. Manα1,3[Manα1,6]Man has two potential conformations in the 500 ns trajectories. ManI was one of the conformations that were fixed at the Core site via the 3-OH and 4-OH dihydroxy. ManII interacted with Glu358 and Ser360 via the ring’s 2-OH and O, which contributed to conformational changes. ManIII was dragged by Asn311 in the Rift site. The Core site and Asn344, Arg345 and Asn362 (Figure 4D) held the second conformation, in which the Asn344, Arg345 andAsn362 formed another pocket, similar to a Valley. In conclusion, DC-SIGN recognized different glycan epitopes at different sites, even if it was only a linker distinction. Meanwhile, we hypothesized two additional sites that could potentially improve the affinity for rationally designed small molecules when paired with DC-SIGN.

**Figure 5:**
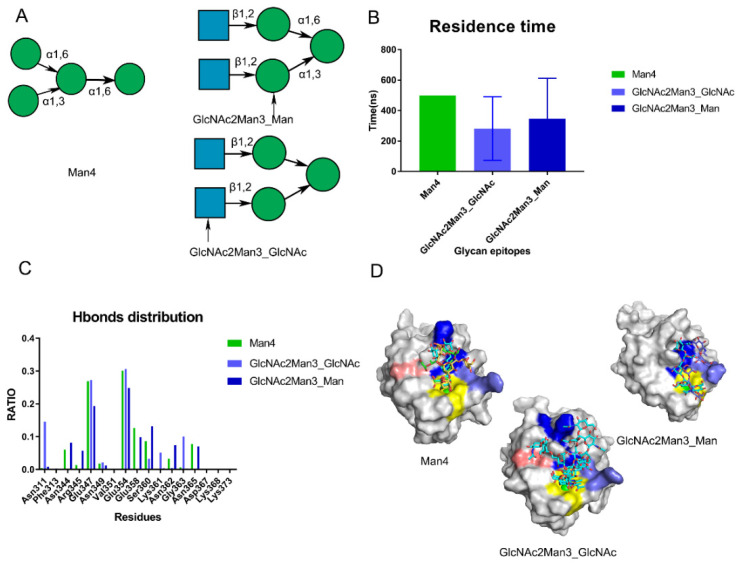
Validation of the proposed sites by performing MD simulations of crystal structures (PDB:1SL4,1K9I). (**A**) the crystal structures on the Man4 (PDB: 1SL4) and GlcNAc2Man3 (PDB: 1K9I). The monosaccharide binding with DC-SIGN’s Ca^2+^ ions in GlcNAc2Man3 crystal structure is indicated by the arrow. (**B**) Residence time along the triple trajectories. (**C**) Hydrogen bond distribution RATIO of these crystal structures along the trajectories. (**D**) Conformations of Man4, GlcNAc2Man3_GlcNAc, GlcNAc2Man3_Man located at different sites along the trajectories. Core site is shown by yellow, Rift site is shown in blue; Valley is shown in purple-blue; Lys368 in pink.

To validate the hypothesized pockets, we performed the Man4 (PDB: 1SL4) and GlcNAc2Man3 (PDB: 1K9I) for 500 ns for triple replicas. Based on the crystal structure, GlcNAc2Man3 was found to possess two states for DC-SIGN (Figure 5A and Figure S4). Based on the residence time, Man4 was found to be more stable than GlcNAc2Man3 in three replicas. (Figure 5B). The distribution of hydrogen bonds showed that the Core site contributed the most hydrogen bonds, emphasizing the relevance of the Core site (Figure 5C). Man4 interacted with the Valley and Rift sites in addition to the Core site. Man4 interacted with Lys368 in some frames along the trajectories. GlcNAc2Man3_Man conformation was dragged by Asn344, Arg345 and Asn362 at the Valley Site for the most time. GlcNAc2Man3_GlcNAc conformation, on the other hand, preferred forming hydrogen bonds with Asn311 and Gly363 at the Rift site. (Figure 5D). Those oligosaccharides were fixed by the Core site and occupied the Valley or Rift sites via its branch. This explained why oligosaccharides had a higher affinity than monosaccharides. Notably, we discovered that GlcNAc2Man3_GlcNAc gradually attached to DC-SIGN from only GlcNAc to whole glycans along the trajectory (Video S1). This could provide a mechanism to explain how DC-SIGN recognizes glycans in reality. In summary, the crystal structures were used to revalidate the potential Valley and Rift sites of DC-SIGN. We hypothesized that the kind of glycan epitopes rather than the precise amino acid sequence determines recognition.

### 3.3. Potential Glycan Epitopes for DC-SIGN on SARS-CoV-2 Spike Protein

**Figure 6:**
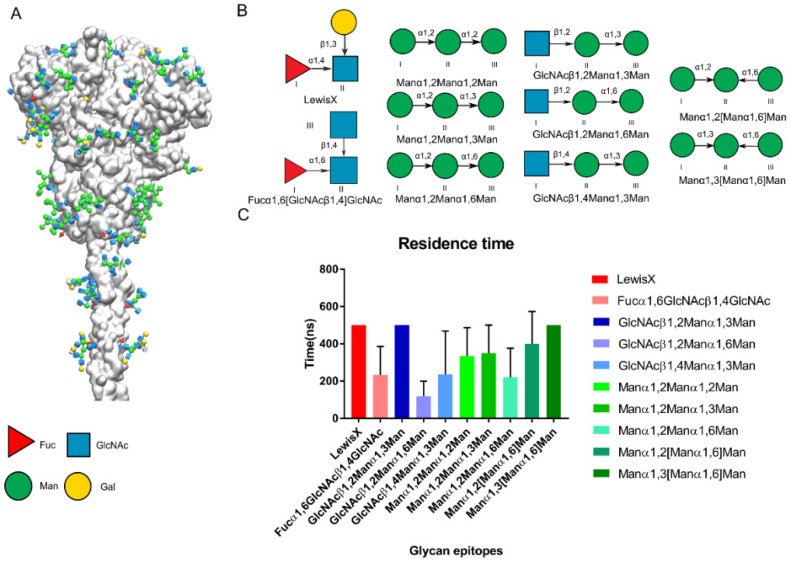
Predicted glycan epitopes on the Spike of SARS-CoV-2 recognized by DC-SIGN. (**A**) First frame of Spike protein [26]. Glycan epitopes on the spike protein shown in the 3D-SNFG [38]. (**B**) The potential epitopes on Spike of SARS-CoV-2 for the DC-SIGN recognition. The antenna epitopes of *N*-type glycostructures on Spike are collected which contained the Fuc, Man, GlcNAc. (**C**) Residence time of potential glycan epitopes of SARS-CoV-2 Spike in triple replicas. Pictures are made by the VMD.

Precise glycoforms of SARS-CoV-2 on the Spike have been identified [15,16,26,50] (Figure 6A). Here, we attempted to predict the glycan epitopes on the Spike that DC-SIGN would recognize based on published papers and our findings above. We collected 10 previously published *N*-type glycan epitopes that potentially bind with DC-SIGN on the SARS-CoV-2 Spike protein (Figure 6B). We constructed initial complex structures using Glide docking and performed MD simulations for a triple replica up to 500 ns. We found that Manα1,2Manα1,3Man and GlcNAcβ1,2Manα1,3Man bound with DC-SIGN were more stable than Manα1,2Manα1,2Man based on residence time. The findings were consistent with those from a recent glycan assay study (Figure 6C) [51]. We inferred that DC-SIGN recognizes glycan epitop50es when glycoforms expose them in a particular conformation. We deduced that other N-glycan sites might be recognized, not limited to Asn149, Asn234 and Asn343.

### 3.4. Natural Glycosides Potentially Act as Antagonists for DC-SIGN

**Figure 7:**
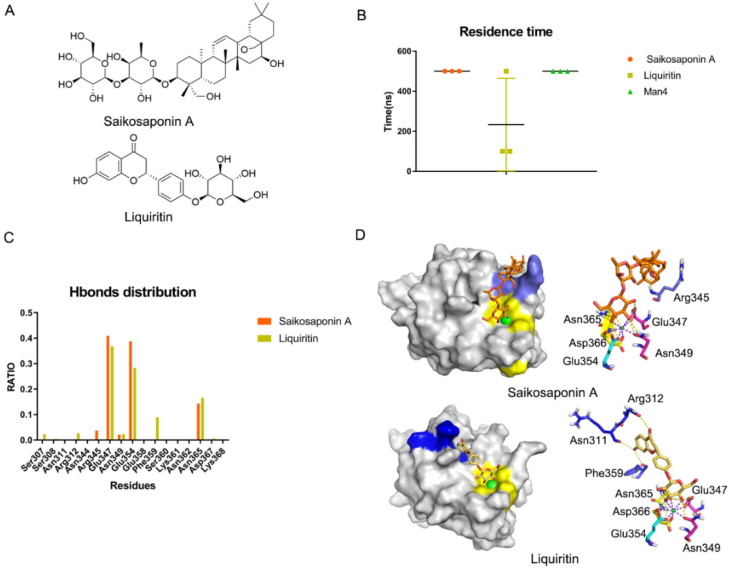
*Saikosaponin A* and *Liquiritin* bind to DC-SIGN in silico. (**A**) Structures of *Saikosaponin A* and *Liquiritin*. (**B**) Residence time of *Saikosaponin A* and *Liquiritin* and Man4 with DC-SIGN in three replicas. (**C**) Hydrogen bonds distribution RATIO about *Saikosaponin A* and *Liquiritin*. Glu347 and Glu354 mostly contributed. (**D**) Binding mode for *Saikosaponin A* and *Liquiritin* in DC-SIGN. Core site, consisted of Glu347, Asn349, Glu354, Asn365 and Asp366, is shown in yellow, Rift site is shown in blue; Valley is shown in purple-blue.

We found that the Core site is critical for recognition and that the Valley and Rift sites may enhance the affinity. Rivipansel is a glycomimetic that inhibits pan-selectins and is now in Phase III clinical trials. Rivipansel has lower hydrophobicity than oligosaccharides, which increases its affinity by 80 times [52]. We hypothesized that reducing the hydrophilicity of the oligosaccharides would enhance their binding affinity in DC-SIGN. Because of the recognition of DC-SIGN and SARS-CoV-2 Spike protein, we hypothesized that natural glycosides can be used as DC-SIGN antagonists to treat COVID-19. In China, Qingfei Paidu decoction is recommended for COVID-19 treatment. It contains 10 natural glycosides, namely *Salidroside*, *Liquiritin*, *Glycyrrhizic acid*, *SaikosaponinA*, *Hesperidin*, *Baicalin*, *Hyperoside*, *Rutin*, *Naringin* and *Prunasin* (Figure S5). Glide docking was used to generate the initial complex structures. This study finding on the binding of molecules with DC-SIGN revealed that (1) they formed coordination bonds with Ca^2+^; (2) they occupied the Valley or the Rift pockets. *Salidroside*, *Glycyrrhizic acid*, *Naringin*, *Liquiritin* and *Saikosaponin A* were found to meet the criteria (Figure S6). To confirm the binding of the five glycosides to DC-SIGN, we performed MD simulations for each of the five systems for a maximum of 500 ns. Based on the residence time, both *Saikosaponin A* and Man4 could bind well to DC-SIGN and be more stable than *Liquiritin* in three replicas (Figure 7B). Following that, we analyzed the key residues as above. It was discovered that *Saikosaponin* A stayed in the Valley pocket while *Liquiritin* preferred the Rift site (Figure 7C,D). Subsequently, we evaluated the binding affinity between the ligands and DC-SIGN via the interface area, which revealed that *Saikosaponin A* > *Liquiritin* > Man4 (Figure S7). A similar result was observed in MM-GBSA, indicating that *Saikosaponin A* and *Liquiritin* may act as DC-SIGN antagonists (Table S2).

## 4. Discussions

Glycosylation is an important and common post-translational modification. Glycans regulate innate immunity via glycan-binding proteins. Here, we investigated how DC-SIGN, a type of glycan-binding protein, recognized potential glycan epitopes. First, we showed that DC-SIGN recognized monosaccharides using MD simulations. Hydrogen bonds, residence time, and MM-GBSA are consistent with the affinity. The monosaccharides were fixed by the EPN motif, NDD motif, and Glu354. Immunity was spurred by Mannose-modified nanomaterials, demonstrating that the monosaccharide could counteract recognition at a certain concentration. Moreover, it was emphasized that monosaccharide binding had the fewest epitopes.

It was also discovered that the affinities of monosaccharides and oligosaccharides varied. Based on previous publications, we hypothesized that glycan epitopes bind to DC-SIGN through different mechanisms. To validate our findings, we performed 500 ns MD simulations for all the reported oligosaccharides. According to the RMSD shown, all of the oligosaccharides could bind to DC-SIGN with a high affinity. Hydrogen bonds distributions revealed that each oligosaccharide had specific residues. Based on those trajectories, we found that the key pocket for recognition, named the Core site, consisted of the EPN motif, NDD motif, and Glu354. Moreover, two additional pockets, one of which is shaped by the EFS Motif and Asn311, may interact with oligosaccharides, similar to a Rift. The other, resembling a Valley, is squeezed by Asn343, Arg344, and Asn362. We believe that the Valley and Rift sites could be potential pockets for enhancing affinity. We performed 500 ns MD simulations on Man4 and GlcNAc2Man3 complex crystal structures to validate the hypothesized pockets and found that the oligosaccharides would use their branch to reach the other pockets in addition to the core site. Furthermore, GlcNAc2Man3_GlcNAc gradually attached to DC-SIGN from GlcNAc to whole glycans, implying a DC-SIGN recognition mechanism. It revealed the mechanism through which DC-SIGN recognized glycans in vivo.

Based on the glycoproteomics of SARS-CoV-2 Spike and our findings, we predicted potential epitopes on Spike that would bind with DC-SIGN. The findings revealed that DC-SIGN can recognize Lewis X, GlcNAcβ1,2Manα1,3Man and Manα1,3[Manα1,6]Man. In addition, it recognized Manα1,2[Manα1,6]Man, Manα1,2Manα1,3Man and Manα1,2Manα1,2Man to a certain extent. We considered that if these glycan epitopes are accessible, DC-SIGN would attach to them, including but not limited to Asn343, Asn234, Asn149 sites.

Similar to Rivipansel, we believed that the Valley and Rift pockets can be used to enhance antagonists’ affinity. In addition, we proposed and validated that natural plant glycosides *Saikosaponin A* and *Liquiritin* would bind to DC-SIGN in silico. The Core site was occupied by their glucopyranose. The subsequent structure used hydroxyl groups to interact with the Valley or Rift site, respectively. The glycosides competed with Spike for binding with DC-SIGN, decreasing the probability of cytokine storm and immune escape. However, more research is needed to determine whether *Saikosaponin A* and *Liquiritin* are pan-antagonists for the C-type lectins family, such as Dectin-1 and P-selectins, or if they are specific for DC-SIGN.

## Figures and Tables

**Table 1:** The MM-GBSA of three monosaccharides binding with DC-SIGN.

Energy Component (kcal/mol)	GlcNAc	Man	Fuc
VDWAALS	−4.5167	−1.1579	1.6843
EEL	−55.6828	−60.8105	−73.6278
EGB	58.3105	58.728	61.582
ESURF	−2.1222	−1.9301	−1.9471
DELTA TOTAL	−4.0112	−5.1706	−12.3085

## Data Availability

Not applicable.

## Supplementary Materials

The following are available online at https://www.mdpi.com/article/10.3390/biom11111586/s1. Figure S1: Binding mode for different monosaccharides (Fuc, Man, GlcNAc) in DC-SIGN, Figure S2: Binding mode for different oligosaccharides in DC-SIGN, Figure S3: Comparison of the residue hydrogen bond distribution RATIO between Manα1,2[Manα1,6]Man and Manα1,3[Manα1,6]Man without considering the core site hydrogen bonding, Figure S4: The conformations of Man4 and GlcNAc2Man3 combined with DC-SIGN in the crystal structure, Figure S5: The structure of the remaining eight natural glycosides in Qingfei Paidu Decoction, except for Saikosaponin A and Liquiritin, Figure S6: Binding mode for different natural product glycosides in DC-SIGN, Figure S7: Interface area between three ligands and DC-SIGN. Table S1: Interface Area in the initial crystal structures, Table S2: The binding free energy of Saikosaponin A and Liquiritin by MM-GBSA. Video S1. The dynamic video of GlcNAc2Man3_GlcNAc in the DC-SIGN protein binding pocket. (Core site in yellow; “Valley” pocket in violet; “Rift” pocket in blue).
