# Supplementary material for: Glycan Epitopes and Potential Glycoside Antagonists of DC-SIGN Involved in COVID-19: In Silico Study
Source: Biomolecules. 2021 Oct 27;11(11):1586. doi: 10.3390/biom11111586 (PMC8615854; doi:10.3390/biom11111586)
Supplement: Supplementary file 1 [file biomolecules-11-01586-s001.zip › biomolecules-1395746-supplementary.pdf]

biomolecules

MDPI

Supplementary

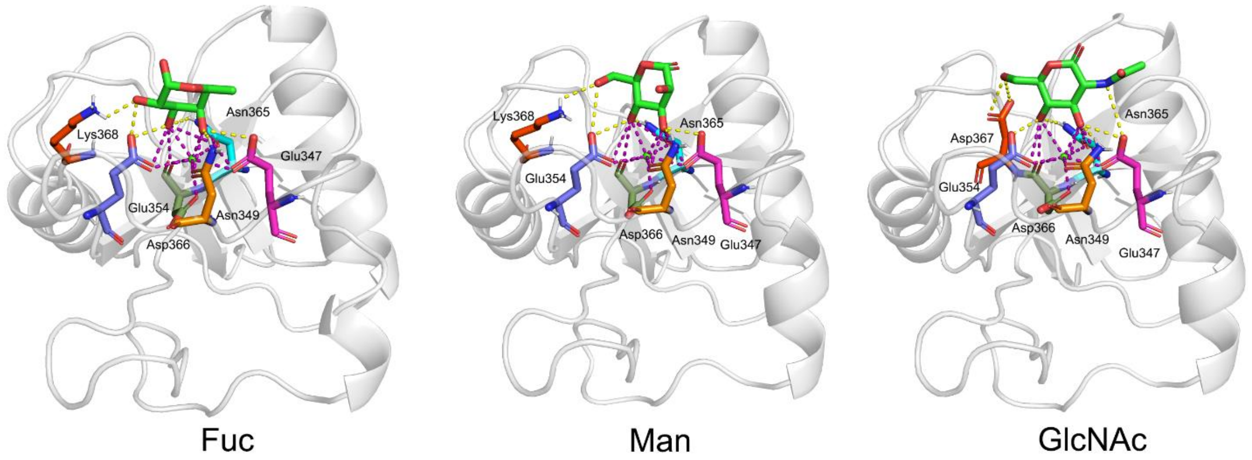

Figure S1. Binding mode for different monosaccharides (Fuc, Man, GlcNAc) in DC-SIGN. It was constructed by Glide V8.9 and worked as initial conformations of MD.

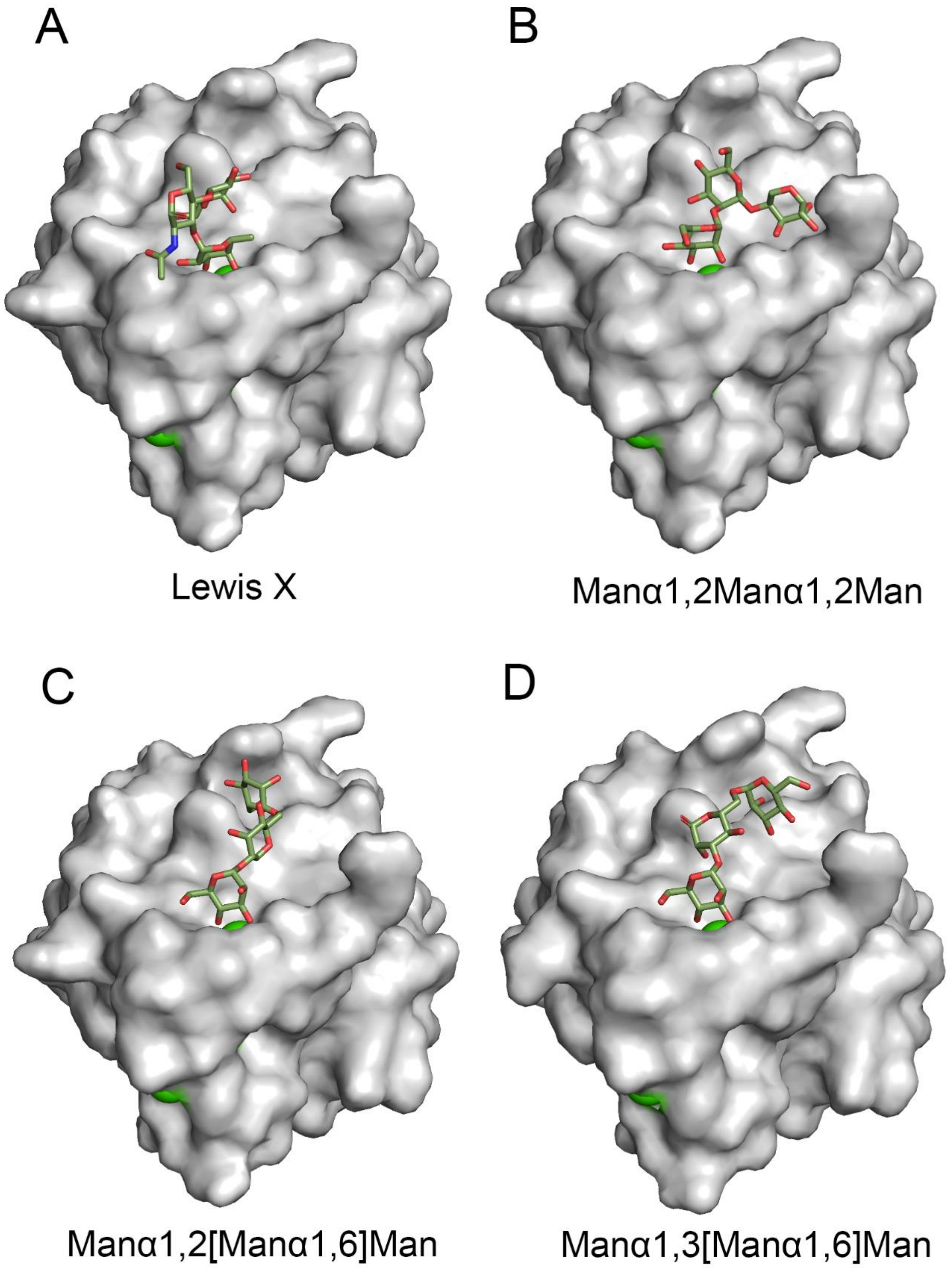

Figure S2. Binding mode for different oligosaccharides in DC-SIGN. A Lewis X; B Manα1,2Manα1,2Man; C Manα1,2[Manα1,6]Man; D Manα1,3[Manα1,6]Man. The binding conformations between published glycan epitopes and DC-SIGN. It was constructed by Glide V8.9 and worked as initial conformations of MD.

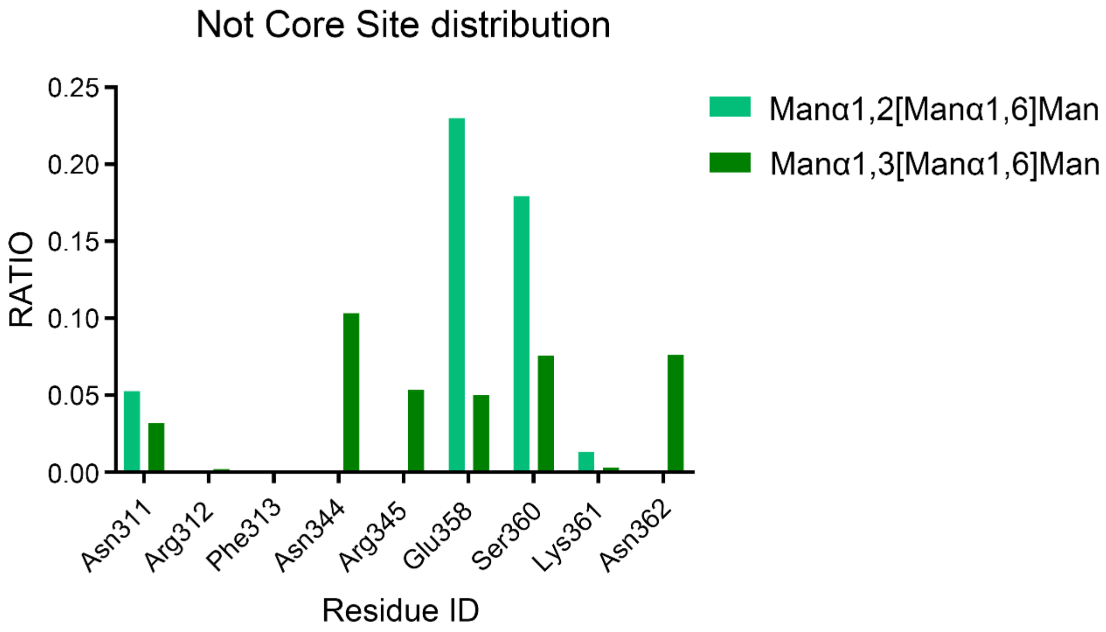

Figure S3. Comparison of the residue hydrogen bond distribution RATIO between Manα1,2[Manα1,6]Man and Manα1,3[Manα1,6]Man without considering the core site hydrogen bonding.

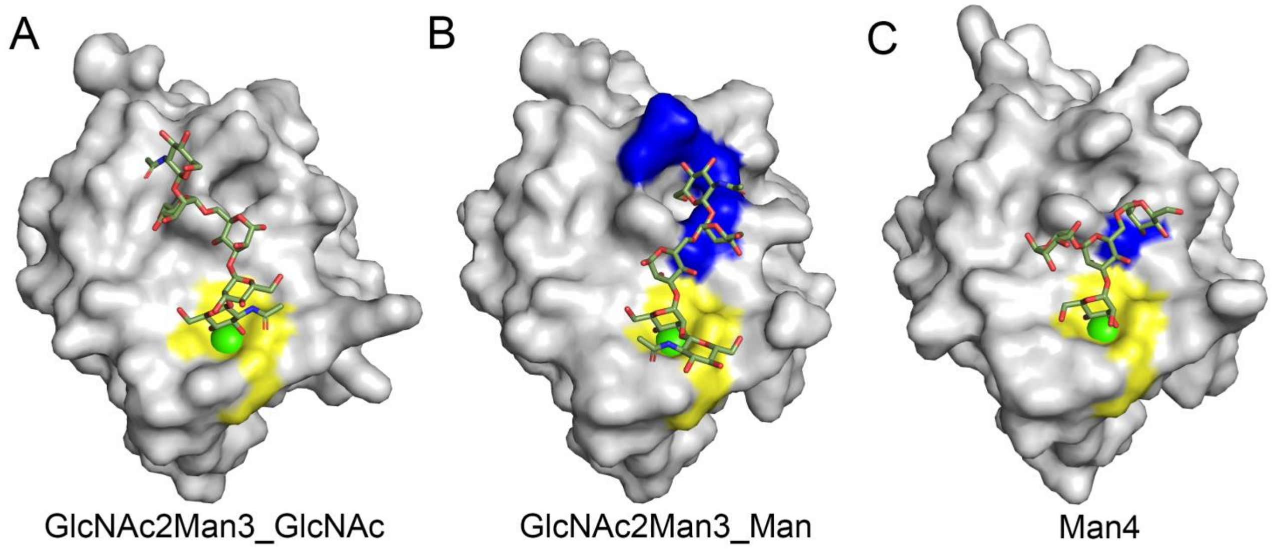

Figure S4. The conformations of Man4 and GlcNAc2Man3 combined with DC-SIGN in the crystal structure. A Conformation of the binding between Ca^{2+} in DC-SIGN and the outermost GlcNAc in GlcNAc2Man3. B Conformation of the binding between Ca^{2+} in DC-SIGN and Man in GlcNAc2Man3. C Conformation and pocket of Man4 combined with DC-SIGN. Core site are shown by yellow, Rift site are shown in blue; (PDB: 1K9I、1SL4).

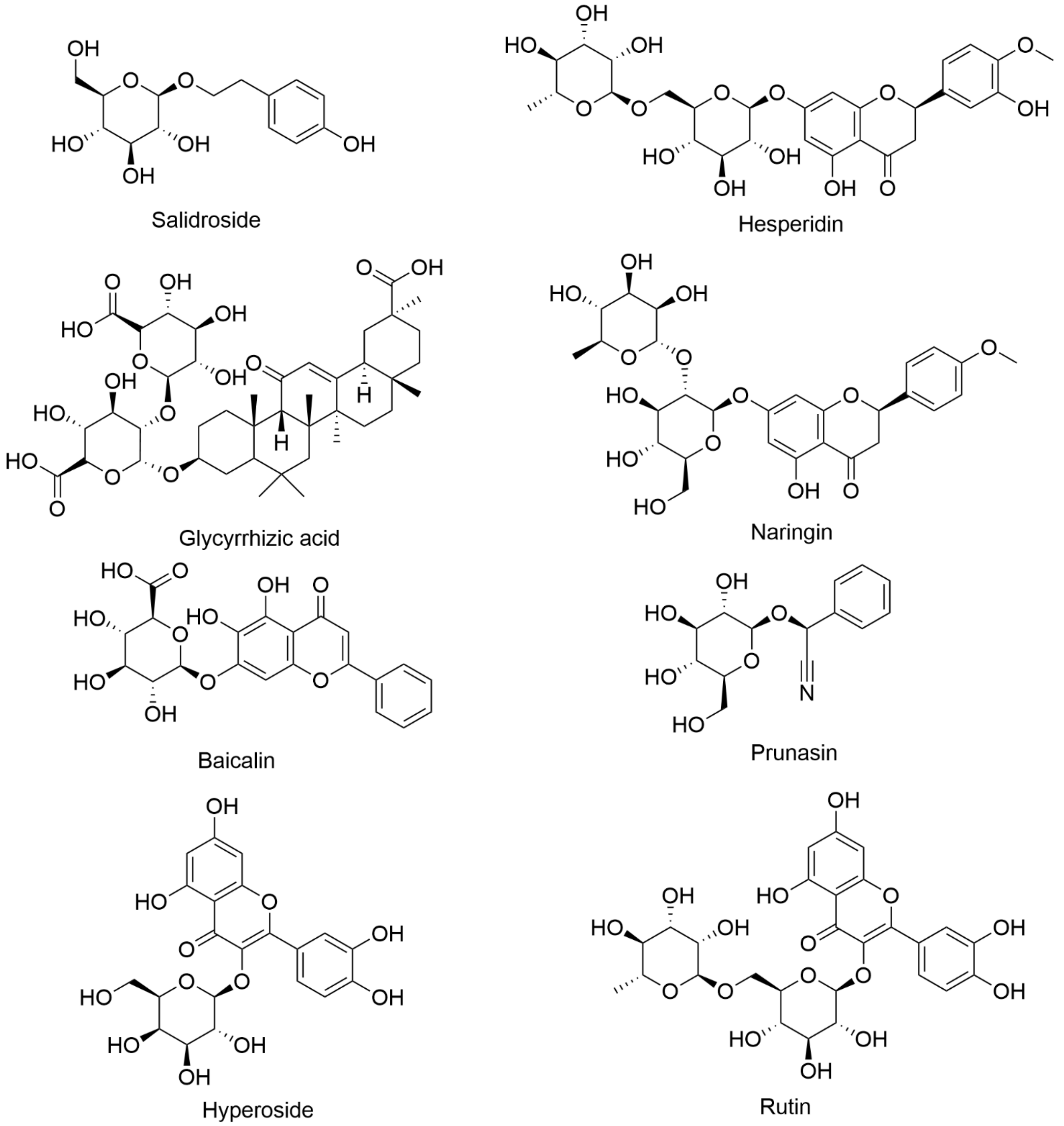

Figure S5. The structure of the remaining eight natural glycosides in Qingfei Paidu Decoction, except for Saikosaponin A and Liquiritin.

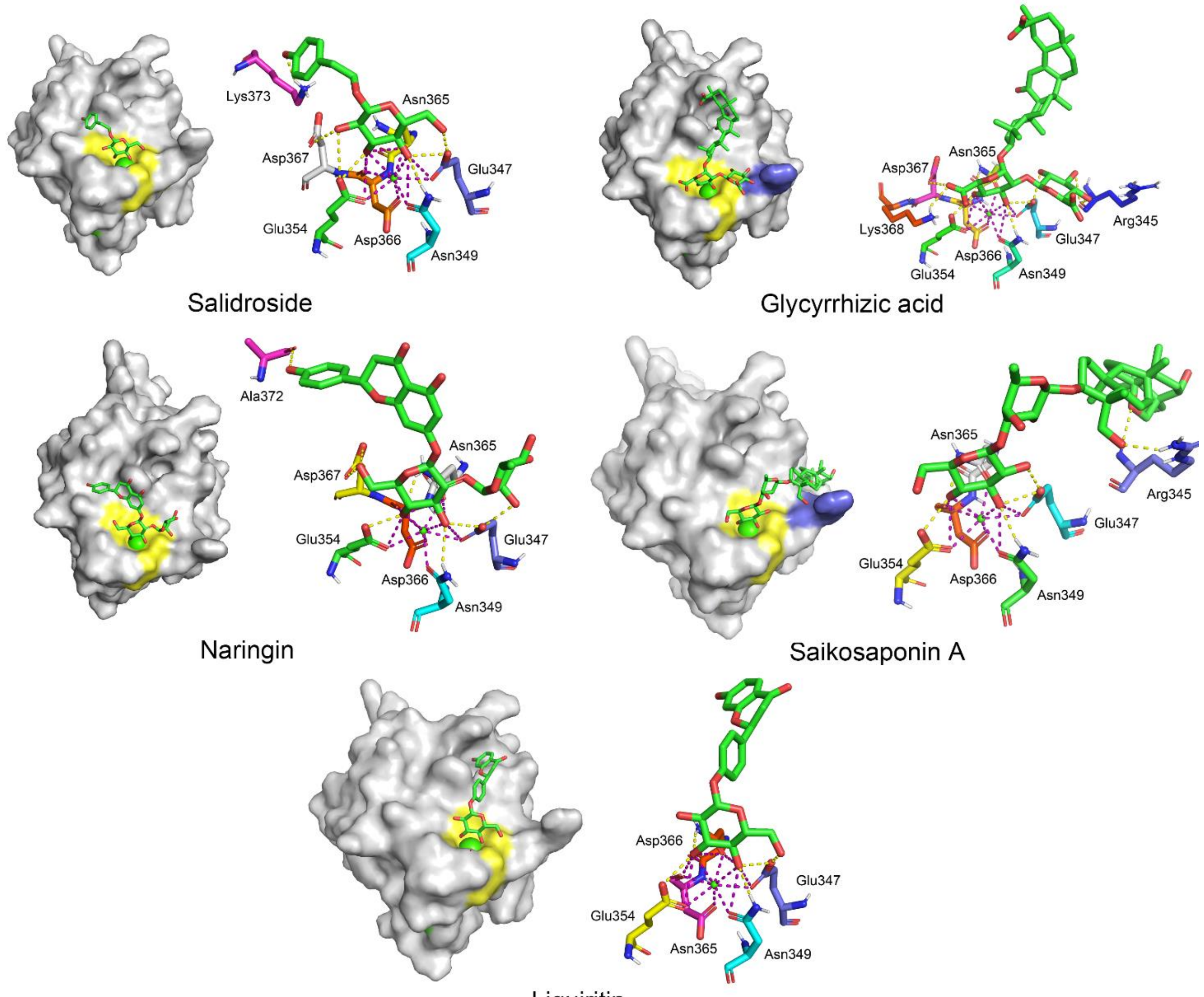

Figure S6. Binding mode for different natural product glycosides in DC-SIGN. It was constructed by Glide V8.9 and worked as initial conformations of MD. The core site is formed by Glu347, Asn349, Glu354, Asn365, Asp366. And the valley site is formed by Asn344, Arg345, and Asn362. Core site are shown in yellow; Valley are shown in purpleblue.

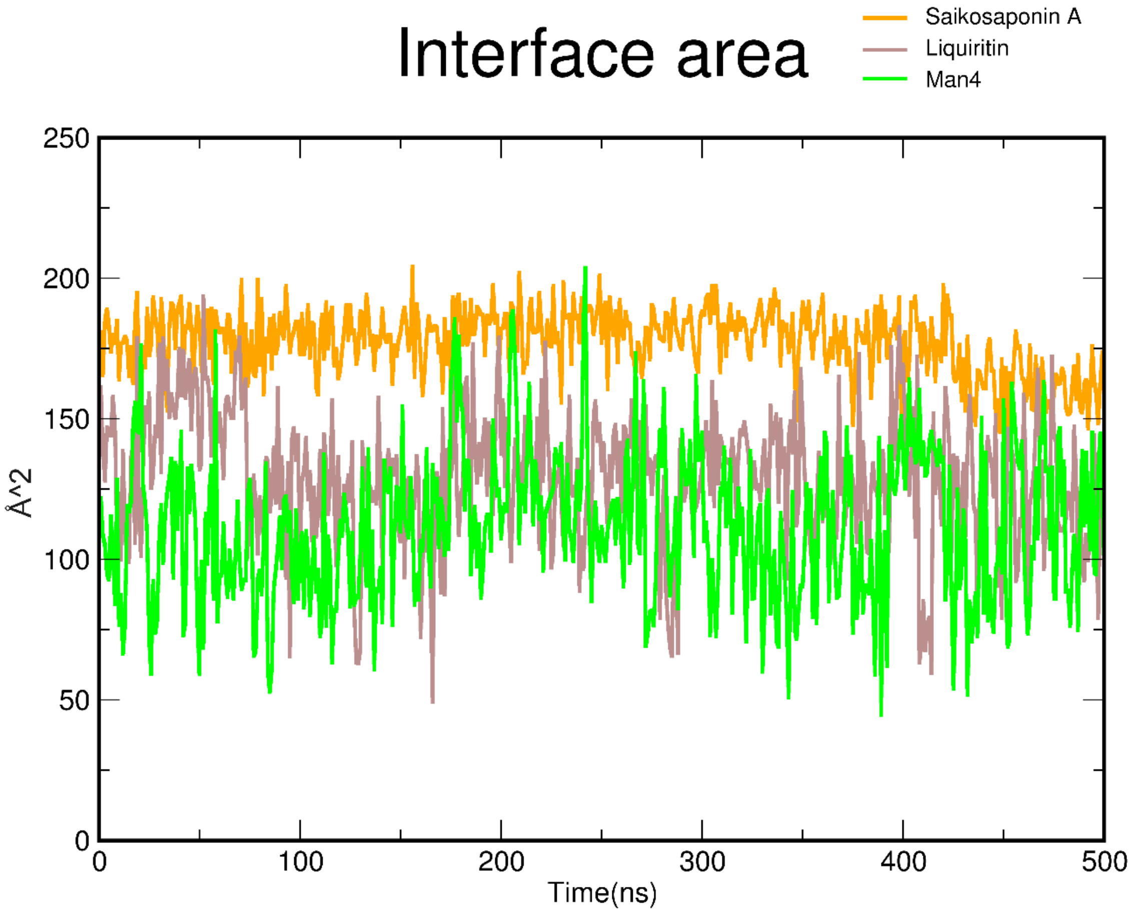

Figure S7. Interface area between three ligands and DC-SIGN. The order of the interface area size is that: Saikosaponin A > Liquiritin > Man4.

Supplementary Table

Table S1. Interface Area in the initial crystal structures.

Compounds (Å^2)	$SASA_{protein}$	$SASA_{ligand}$	$SASA_{complex}$	Interface Area
Fuc	6338.4593	158.5075	6374.8992	61.0338
Man	6270.1932	161.9391	6315.0913	58.5205
GlcNAc	5993.6415	211.8934	6108.5823	48.4763
Manα1,2Man	6270.1932	271.1824	6329.9183	105.72865
Lewis X	6338.4593	398.3933	6526.1542	105.3492
Man4	6286.1805	516.6714	6586.8111	108.0204
GlcNAc2Man3_Man	5926.7745	704.6171	6222.5675	204.41205
GlcNAc2Man3_GlcNAc	5992.8016	704.6171	6587.7838	54.81745

* Solvable accessible surface area (SASA).

Table S2. The binding free energy of Saikosaponin A and Liquiritin by MM-GBSA.

Energy Component (kcal/mol)	Saikosaponin A	Liquiritin	Man4
VDWAALS	-29.4311	-13.2193	-23.0099
EEL	-42.2283	-66.3147	-54.8062
EGB	56.2091	68.4339	73.6976
ESURF	-4.5279	-3.5974	-4.5727
DELTA TOTAL	-19.9782	-14.6975	-8.6912

Supplementary Videos

Video S1. The dynamic video of GlcNAc2Man3_GlcNAc in the DC-SIGN protein binding pocket. (Core site in yellow; "Valley" pocket in violet; "Rift" pocket in blue).
